# Does Language Make a Difference? A Study of Language Dominance and Inhibitory Control

**DOI:** 10.3389/fpsyg.2021.648100

**Published:** 2021-07-29

**Authors:** Ashley Marie Salwei, Beatriz de Diego-Lázaro

**Affiliations:** ^1^Department of Clinical Psychology, Midwestern University, Glendale, AZ, United States; ^2^Department of Speech and Language Pathology, Midwestern University, Glendale, AZ, United States

**Keywords:** bilingualism, inhibitory control, children, language dominance, vocabulary

## Abstract

Although extensive research has been done to compare monolingual and bilingual children’s executive function, there are fewer studies that look at the relation between bilingual children’s languages and executive function. The purpose of this study was two-fold; first, to compare inhibitory control (executive function) in monolingual and bilingual children and second, to determine what vocabulary measure (dominant vs. non-dominant language) was related to inhibitory control in bilingual children. Twenty monolingual (English) and 20 bilingual (English-Spanish) children between the ages of 8 and 12 completed a vocabulary test (in English and Spanish) and an inhibitory control task (the flanker task). Analysis of Covariances (ANCOVAs) revealed no significant differences between monolingual and bilingual children in reaction time (RT) or accuracy in the flanker task after controlling for maternal education. Partial correlations controlling for age showed that English expressive vocabulary (dominant language), but not Spanish, was positively correlated with inhibitory control (larger vocabulary and better inhibitory control), suggesting that bilingual children may use their dominant language to self-regulate over their non-dominant language, increasing the inhibitory control associated to the dominant language.

## Introduction

Language and executive functions are two distinct brain tasks, yet they are related and difficult to separate, creating a debate about what function influences the other; does cognition precede language, does language drive cognition, or are they bidirectionally influenced? Not only is there a debate about how these tasks are related, but we also have limited information about how these tasks change if the individual is monolingual vs. bilingual. Extensive past research has focused on identifying differences in executive function between monolingual and bilingual children with inconclusive results; some suggest there is a bilingual advantage (e.g., Carlson and Meltzoff, 2008; Martin-Rhee and Bialystok, 2008; Adesope et al., 2010; Foy and Mann, 2014), while others propose there is no significant advantage for bilinguals (e.g., Antón et al., 2014; Duñabeitia et al., 2014; Ross and Melinger, 2017; Arizmendi et al., 2018). Yet, there are fewer studies that look at the relation between bilingual children’s languages and executive function (e.g., Poulin-Dubois et al., 2011; Crivello et al., 2016; Kuzyk et al., 2020). The purpose of this study was two-fold; first, to compare inhibitory control (executive function) in monolingual and bilingual children and second, to determine what vocabulary measure (dominant vs. non-dominant language) was related to inhibitory control in bilingual children.

## Inhibitory Control (Monolingual Vs. Bilingual Children)

Inhibitory control, an executive function, allows an individual to control their impulses and helps increase focus on relevant information by inhibiting irrelevant information. The Inhibitory Control Model (Green, 1998) proposes that bilinguals have a cognitive advantage over monolinguals because bilinguals continually shift between two languages, in principle, strengthening their inhibitory control. This model implies that bilinguals have more cognitive flexibility than monolinguals, and this cognitive flexibility is translated into a cognitive advantage across many domains, linguistic and non-linguistic (Liu et al., 2016).

Some researchers have found a bilingual advantage on executive function and working memory, particularly in balanced bilinguals, those that show similar proficiency and use of both languages (e.g., Bialystok and Majumder, 1998; Adesope et al., 2010; Yow and Li, 2015; Rosselli et al., 2016; Kalia et al., 2019). For example, Carlson and Meltzoff (2008) assessed the vocabulary, executive function, and non-verbal cognition of 50 kindergarten children classified in three groups: the simultaneous bilingual group (12 children exposed to Spanish and English since birth), the immersion group (21 children attending a language immersion school where half the day was in English and the other half in Spanish), and the control group (17 English monolingual children attending traditional kindergarten programs). The researchers found that the simultaneous bilingual children outperformed both groups on tasks of executive function. Similarly, Kalia et al. studied vocabulary and inhibitory control in 61 Spanish-English dual-language learners and 55 English monolingual kindergarten to third grade children. They found that, the dual-language learners outperformed the monolingual children on measures of inhibitory control.

Other researchers have found no evidence of a bilingual advantage on executive function tasks (e.g., Antón et al., 2014; Duñabeitia et al., 2014; Ross and Melinger, 2017; Arizmendi et al., 2018). For example, Antón et al. assessed executive function (Attentional Network Test and the flanker task) in 180 elementary-aged bilingual children (Basque and Spanish) and 180 monolingual children (Spanish). The researchers found no significant differences between the groups and thus the bilingual advantage was not supported. Similarly, Arizmendi et al. assessed executive function (including inhibitory control) in 167 monolingual (English) and 80 bilingual (Spanish-English) second graders. The results revealed no differences between monolingual and bilingual children. The authors sampled a group of bilinguals who attended English-only education and they suggested that in order for the bilingual children to demonstrate an advantage, it is possible that they would have to regularly switch between the two languages; an opportunity that may not be present given their predominantly English exposure at school. Taken together, research remains inconclusive on whether an executive function advantage truly exists in bilingual children and what factors may contribute to it.

## Relation Between Inhibitory Control and Language

### Language Dominance

In order to study the relation between inhibitory control and language in bilingual children, we first need to define language dominance. The dominant language is the language most frequently used by an individual and the one with higher proficiency levels (Unsworth, 2016; Treffers-Daller, 2019). Language dominance is determined by the amount of exposure to that language and its use, however, there are external factors that affect language exposure and use that could indirectly affect language dominance such as language status. The majority language or the language used more frequently in the community (English in the United States) tends to be favored over the minority language (Spanish; Paradis and Nicoladis, 2007). By middle childhood, Spanish-speaking children in the United States tend to move from Spanish to English dominance (Kohnert and Bates, 2002; Buac et al., 2014).

Language has different domains (e.g., vocabulary, phonology, or grammar) and therefore there are different ways to define language dominance depending on the language domain. Typically, in order to establish language dominance in bilingual children, researchers measure language exposure using caregivers’ questionnaires or asking older children to report the amount of exposure and use in a particular language (Bedore et al., 2012; Unsworth, 2016). In addition, proficiency tasks, such as standardized tests, can be also used to define language dominance in a particular language domain. Previous studies have shown that direct measures of language proficiency and indirect measures of language use are typically strongly correlated (e.g., Bedore et al., 2012; Unsworth, 2016; but see Lim et al., 2008).

Language mediates information processing, learning, and memory (see Verbal Mediation Hypothesis, Diaz and Klingler, 1991). Bilingual individuals use the dominant language more frequently than the non-dominant language when self-regulating (private speech) and processing new information; in other words, they “think” more frequently in the dominant language than in the non-dominant language (e.g., Francis and Baca, 2014; Jiménez Jiménez, 2015; Sawyer, 2016). Considering this, it will not be surprising to find stronger relations between bilinguals’ dominant language and executive function over the non-dominant language, as they use their dominant language more frequently when mentally manipulating information in their private speech. While there is some evidence in this direction (e.g., Kalia et al., 2019; but see Nicoladis et al., 2018), previous studies on bilingualism and executive function have focused on identifying differences in executive function between monolinguals and bilinguals (Carlson and Meltzoff, 2008; Adesope et al., 2010; Antón et al., 2014) or establishing directionality (what comes first) between language and executive function (e.g., Bohlmann et al., 2015; Blom, 2019), rather than looking at the relations between the dominant and non-dominant language and executive function.

### Inhibitory Control and Language in Bilingual Children

Previous studies in monolingual children have shown evidence of both unidirectional and bidirectional relations between language and executive function (including inhibitory control). While some authors suggest that executive function comes first and predicts language outcomes (e.g., Weiland et al., 2014; White et al., 2017; Ekerim and Selcuk, 2018; Larson et al., 2020) others suggest just the opposite, that language ability comes first and predicts inhibitory control (e.g., Fuhs and Day, 2011; Kuhn et al., 2016; Botting et al., 2017). Bidirectional relations have been also reported in previous studies in which language and executive function influence each other equally (e.g., Kaefer and Neuman, 2013; Bohlmann et al., 2015; Slot and von Suchodoletz, 2018). Likewise, studies in bilingual children have found evidence of unidirectional and bidirectional relations between inhibitory control and language outcomes.

#### Unidirectional Approach

Unidirectional relations have been explored in bilingual children, looking at the relation between the dominant language and inhibitory control. For example, Gangopadhyay et al. (2019) looked at the relation between inhibition and lexical processing in monolingual (English) and simultaneous bilingual (English-Spanish) children by having them complete a Lexical Decision Task (LDT) in English and two nonverbal inhibitory control tasks. The researchers collected data at two separate time points 1 year apart. The authors found that both groups had similar processing speed and they found that LDT performance in Year 1 predicted inhibition in Year 2 for both groups, but the reverse relation was not observed.

Other researchers have proposed a unidirectional approach suggesting just the opposite finding; that executive function predicts language outcomes (White et al., 2017; Blom, 2019). For example, Blom (2019) investigated the relation between bilingual children’s inhibitory control and vocabulary in both the dominant (Dutch) and the non-dominant (Turkish, Tarifit) languages. The researchers collected data three times a year for 2 years starting when the 69 participants were 5 or 6 years old. The researchers found that executive function did not predict vocabulary in the non-dominant language but did predict vocabulary in the dominant language. Blom concluded that attention allowed individuals to learn over time by focusing on what is relevant and ignoring what is not. Therefore, individuals with better attention skills will process information better and have a greater vocabulary. Similarly, Escobar et al. (2018) studied the relation between language and inhibitory control in school-aged (8-year-old) bilingual and monolingual children by having them complete English verbal fluency tasks and two measures of inhibitory control. The authors found that inhibitory control was a predictor of verbal fluency in both monolingual and bilingual children.

#### Bidirectional Approach

A bidirectional approach has been proposed in bilingual individuals suggesting that both language and executive function influence each other equally (Bohlmann et al., 2015; Slot and von Suchodoletz, 2018; Kalia et al., 2019). Bohlman et al. studied the relation between English expressive vocabulary and self-regulation skills in 73 English monolingual and 177 English-Spanish preschoolers. The results indicated a bidirectional relation between self-regulation and English expressive vocabulary. Similarly, Kalia et al. reported significant correlations between English, but not Spanish, expressive vocabulary and inhibitory control in 26 English-Spanish learners (5–10 years old; balanced bilinguals), in which the larger the English expressive vocabulary the higher the inhibitory control (accuracy in card sorting and Stroop tasks). These results indicate that language and inhibitory control probably influence each other.

Taken together, regardless of directionality, it seems that language (vocabulary size) and inhibitory control are correlated, in which the higher the vocabulary the better the inhibitory control. Questions remain about how the relation between vocabulary and inhibitory control is expressed in bilingual children taking into account language dominance. In this study, we used a sample of English-dominant bilingual children to observe what vocabulary measure (dominant vs. non-dominant language) was related to inhibitory control.

### Purpose

The purpose of this study was two-fold; first, to compare inhibitory control in monolingual and bilingual children and second, to determine what vocabulary measure (dominant vs. non-dominant language) was related to inhibitory control in bilingual children. Questions and hypothesis are as follows:

1. Do monolingual and bilingual children differ in inhibitory control? We hypothesized that if experience controlling two languages enhances inhibitory control, then bilingual children will exhibit greater inhibitory control than monolinguals.
2. What vocabulary measure (dominant vs. non-dominant language) is related to inhibitory control in bilingual children? We hypothesized that if bilingual children use their dominant language more often than the non-dominant language to self-regulate and process new information, then vocabulary in the dominant language will be correlated with inhibitory control more strongly than vocabulary in the non-dominant language.

## Materials and Methods

### Participants

Participants came from a larger study on word learning that included monolingual and bilingual children with and without hearing loss (de Diego-Lázaro et al., 2021). This study was approved by the Institutional Board Review at Arizona State University. Prior to participation, child assent and parental consent were obtained.

Participant criteria for the current study included: (a) monolingual English or bilingual English-Spanish children between 8 and 12 years of age, (b) normal hearing (<20 dB in 500, 1,000, 2,000, and 4,000 Hz) and typical development (per caregiver report), and (c) input and output in the non-dominant language greater than 30% of the weekly time (for bilingual children). Three bilingual participants did not meet inclusion criteria and were removed from the analyses because their weekly Spanish use was below 30% and they were unable to use Spanish expressively. Monolingual children were not exposed to a second language at home and they were not enrolled in bilingual education. In total, 40 children were selected for the study: 20 monolinguals (English) and 20 bilinguals (English-Spanish). Table 1 shows the participants’ characteristics by group (monolingual and bilingual). While gender and age were similar between groups, monolingual children had mothers with higher educational levels than bilingual children.

**Table 1:** Participants’ characteristics.

Characteristics	Monolingual (*n* = 20)	Bilingual (*n* = 20)	*X* ^2^
Gender (male)	55%	45%	0.400
Age	10.9 (1.5)	11.6 (1)	0.620^a^
Maternal education^b^			14.54^**^
College/University	85%	25%	
High school or less	15%	75%	
High school	15%	35%	
Elementary	0%	40%	

% or mean and (SD).

a Independent sample *t*-test.

b High school or less vs. more than high school.

** *p* < 0.01.

Table 2 outlines the characteristics of the bilingual group. Most of the bilingual participants learned Spanish at home and English at school, although 45% reported sole English use when communicating with siblings in the home. With the exception of one child born in Mexico, all participants were born in the United States. During the week, participants reported being exposed to Spanish 51% of the time and used Spanish 50% of the time. This balanced exposure and use between English and Spanish would suggest no language preference; however, the participants demonstrated an English language preference when interacting with the bilingual investigators.

**Table 2:** Bilingual participants’ characteristics.

Characteristics	Bilingual(*n* = 20)
English acquisition age (years)^a^	3.5 (1.5)
Spanish acquisition age (years)^a^	1.2 (1.1)
English-only school (years)	5.9 (3.4)
Spanish-only school (years)	0.6 (1.9)
Dual-language school (years)	1.4 (3.0)
English self-proficiency	
Understanding	4.7 (0.4)
Speaking	4.7 (0.4)
Reading	4.6 (0.6)
Spanish self-proficiency	
Understanding	4.1 (0.8)
Speaking	4.0 (0.8)
Reading	3.2 (1.4)
Speaking preference	
English	20%
Spanish	15%
No preference	65%
Reading preference	
English	65%
Spanish	5%
No preference	30%

% or mean and (SD). Self-proficiency scale = 1 “very poor” to 5 “very good.”

a Age around first word (expressively).

### Measures

#### Caregiver and Child Language Questionnaire

We interviewed children and caregivers in their dominant language to collect demographic information (i.e., age, maternal education level, and family composition), language history, and language exposure/use. We used the Bilingual Input-Output Survey (BIOS) from the Bilingual English–Spanish Assessment (BESA; Peña et al., 2014) to calculate the percentage of input/output in each language. Additionally, the bilingual children expressed their language preferences for speaking and reading, and they filled out a rating scale (1 “very poor” to 5 “very good”) on their language proficiency (see Table 2). Some of the questions asked in the interview were adapted from the Language Experience and Proficiency Questionnaire (LEAP-Q; Marian et al., 2007) and from the BESA (Peña et al., 2014).

#### Expressive Vocabulary

We measured bilingual expressive vocabulary using the bilingual Expressive One-Word Picture Vocabulary Test-Bilingual Version (EOWPVT-IV; Martin and Brownell, 2010). This test was created and normed on bilingual children from the United States and contains 180 items. We began presenting the items at word one and stopped when children missed six consecutive words (ceiling), as recommended in the manual. The test was presented in PowerPoint and each slide contained a picture children had to name using one word. The test was used twice, once in English and once in Spanish, counterbalanced. The instructions were given in English and Spanish (respectively) for bilinguals and in English for monolinguals. Given that Spanish is a common language in the United States, the monolingual participants were also given the opportunity to name pictures in Spanish. Overall, the test provided an estimate of the expressive words the participants knew in English, Spanish, and in total (English + Spanish). We used raw scores instead of standard scores because the bilingual EOWPVT-VI does not offer standard scores for each language separately. This test only provides a global conceptual score (i.e., concepts across languages). Raw scores in each language were used to establish language dominance.

#### Inhibitory Control

The flanker task measures inhibitory control because it assesses the ability to suppress responses that are inappropriate within a particular context (Eriksen, 1995). In this task, children indicated the direction of an arrow presented on the screen by pressing the right or left key. The central arrow was flanked by arrows in the same direction (congruent trials: →→→→→), opposite direction (incongruent trials: →→←→→), or no arrows (neutral trials: __→__). The task was presented on a computer monitor using Psychopy software (Peirce, 2007). Participants had 10 practice trials with feedback in which they were instructed to pay attention to the target arrow and indicate its direction (left or right) as quickly as possible using the keyboard. Children were required to achieve >80% accuracy in the practice trials to continue to the experimental task to ensure that they understood the instructions. No children were excluded from this task. Instructions for bilingual children were provided in English and in Spanish. A fixation point was presented throughout the task in the center of the screen. Once children were familiarized with the task, they were presented with 48 trials; 16 congruent, 16 incongruent, and 16 neutral trials in random order.

### Analyses

Inhibitory control was measured by accuracy in the incongruent trials and by subtracting the congruent reaction time (RT) from the incongruent RT in correct trials only. To assess whether there was a difference in inhibitory control between monolingual and bilingual children, we performed two Analysis of Covariance (ANCOVA) with language experience as the independent variable (monolingual vs. bilingual), maternal education (high school or less vs. more than high school) as a covariate, and inhibitory control RT (incongruent-congruent) and accuracy (incongruent trials) as dependent variables, respectively.

To identify what vocabulary measure (dominant vs. non-dominant language) was related to inhibitory control, we first ran bivariate correlations between demographic, vocabulary, and inhibitory control measures. Then, we ran partial correlations between English expressive vocabulary and inhibitory control measures controlling for age.

## Results

### Descriptive Data

Table 3 shows descriptive data for the vocabulary and inhibitory control measures by group. Monolingual children had a larger English expressive vocabulary but a smaller Spanish vocabulary than the bilingual children. The monolingual and bilingual children showed similar reaction time and accuracy in the three conditions of the flanker task (neutral, congruent, and incongruent trials).

**Table 3:** Vocabulary and inhibitory control measures by group.

Variable	Monolingual (*n* = 20)	Bilingual (*n* = 20)	*t*
Expressive English^a^	122.5 (12.7)	110.3 (12.1)	3.10^*^
Expressive Spanish^a^	0.5 (0.2)	75.0 (28.0)	−11.74^**^
Congruent RT	715 (114)	727 (169)	−0.284
Incongruent RT	832 (146)	891 (202)	−1.06
Neutral RT	682 (109)	718 (165)	−0.812
Congruent %	99 (3)	99 (1)	−0.872
Incongruent %	94 (7)	92 (7)	0.726
Neutral %	99 (2)	100 (1)	−0.588

Mean and (SD). RT, Reaction time in milliseconds for correct trials.

a Expressive vocabulary raw scores from the Expressive One-Word Picture Vocabulary Test-Bilingual Version (EOWPVT-IV), maximum 180.

* *p* < 0.05;

** *p* < 0.01.

Language dominance for bilingual children was established considering proficiency measures (direct and self-reported measures). Direct measures of vocabulary in each language showed that all children had higher English than Spanish vocabularies [*t*(19) = 5.39, *p* < 0.001]. Self-reported proficiency measures showed that, on average, children perceived that they were able to understand [*t*(19) = 2.60, *p* = 0.017], speak [*t*(19) = 3.11, *p* = 0.006], and read [*t*(19) = 4.07, *p* = 0.001] in English better than in Spanish (see Table 2 for means and SDs). Despite the fact that language exposure and use were balanced in this group of bilingual children, proficiency measures showed a clear preference for English over Spanish. Since this study investigates the relation between vocabulary and inhibitory control, we considered proficiency as a more accurate and direct measure to establish vocabulary dominance than language exposure. Therefore, bilingual children were English-dominant for vocabulary.

### Monolingual vs. Bilingual Inhibitory Control

**Figure 1:**
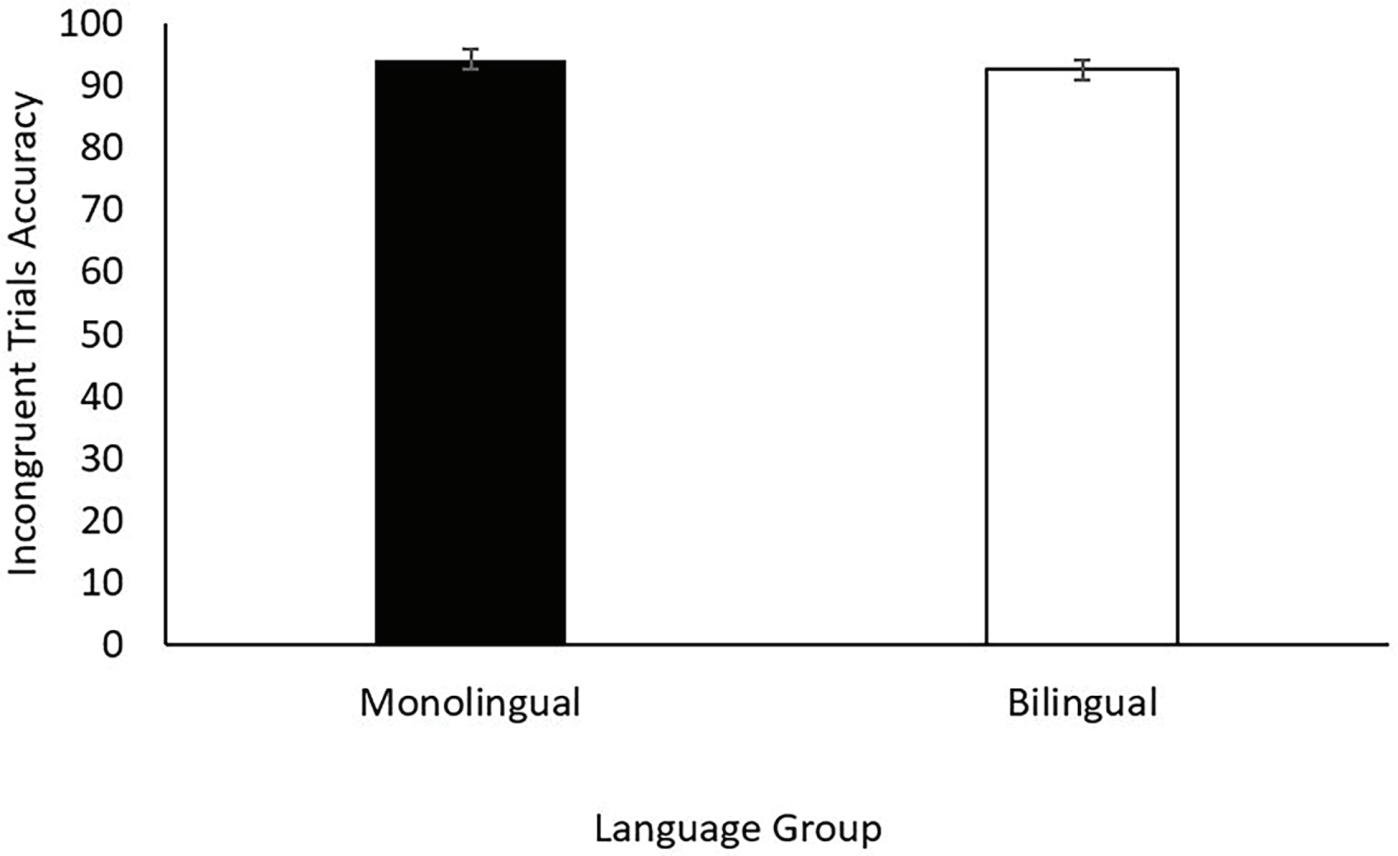
Mean and SE for reaction time (RT) in milliseconds (incongruent-congruent trials) in the flanker task (inhibitory control).

**Figure 2:**
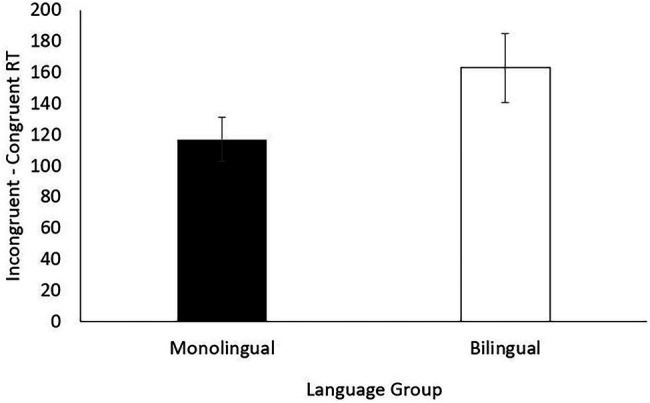
Mean and SE for incongruent trials accuracy in the flanker task (inhibitory control).

Figures 1, 2 show reaction time (incongruent-congruent trials) and accuracy (incongruent trials) in the flanker task for monolingual and bilingual children. ANCOVAs controlling for maternal education revealed no significant differences neither in reaction time [*F* (1, 37) = 0.200, *p* = 0.657, *η*^2^ = 0.005] nor in accuracy [*F* (1, 37) = 0.012, *p* = 0.913, *η*^2^ < 0.001], suggesting that bilingual and monolingual children showed similar inhibitory control.

### Vocabulary Dominance and Inhibitory Control

Table 4 shows the correlations between demographic, vocabulary, and inhibitory control measures for monolingual children. Age was significantly correlated with English expressive vocabulary in which the older the child the larger their vocabulary. No significant correlations were found between English expressive vocabulary and inhibitory control measures.

**Table 4:** Correlation matrix between demographic, vocabulary, and inhibitory control measures in the monolingual children (*n* = 20).

Variable	Maternal education	English vocabulary	Spanish vocabulary	Incongruent – congruent RT	Incongruent %
Age	0.10	0.41^*^	0.24	−0.27	0.28
Maternal education		0.02	0.10	−0.09	−0.11
English vocabulary			−0.47^*^	−0.38	0.09
Incongruent – congruent RT					0.04

RT, Reaction time in milliseconds for correct trials.

* *p* < 0.05.

Table 5 shows the correlations between demographic variables, vocabulary, and inhibitory control measures for bilingual children. Age was significantly correlated with English expressive vocabulary in which the older the child the larger their vocabulary. Age was also correlated with the inhibitory control measures in which the older the child the shorter the reaction time, and the higher the accuracy in the incongruent trials of the flanker task. Given the significant correlations between age and English vocabulary, and between age and inhibitory control measures, we ran partial correlations between English vocabulary and inhibitory control controlling for age. English, but not Spanish expressive vocabulary was correlated with reaction time [*r*(17) = −0.20, *p* = 0.043] and accuracy [*r*(17) = 0.51, *p* = 0.013] in which the larger the vocabulary the shorter the reaction time and the higher the accuracy in the incongruent trials. Finally, reaction time and incongruent trials’ accuracy were negatively correlated in which the shorter the reaction time, the higher the accuracy.

**Table 5:** Correlation matrix between demographic, vocabulary, and inhibitory control measures in the bilingual children (*n* = 20).

Variable	Maternal education	English vocabulary	Spanish vocabulary	Incongruent – congruent RT	Incongruent %
Age	0.01	0.73^**^	−0.09	−0.51^*^	0.62^**^
Maternal education		0.35	−0.21	−0.36	0.33
English vocabulary			0.10	−0.50^*^	0.73^**^
Spanish vocabulary				0.09	0.24
Incongruent – congruent RT					−0.50^*^

RT, Reaction time in milliseconds for correct trials.

* *p* < 0.05;

** *p* < 0.01.

## Discussion

The purpose of this study was two-fold; first, to compare inhibitory control (executive function) in monolingual and bilingual children and second, to determine what vocabulary measure (dominant vs. non-dominant language) was related to inhibitory control in bilingual children. The results showed that monolingual and bilingual children did not differ in inhibitory control as measured by the flanker task. English expressive vocabulary (dominant language), but not Spanish, was significantly correlated with inhibitory control, suggesting that bilingual children may use their dominant language to self-regulate over their non-dominant language while participating in a task that requires inhibition.

### Monolingual vs. Bilingual Inhibitory Control

Contrary to our first hypothesis, we did not find that bilingual children outperformed monolingual children in inhibitory control (reaction time and accuracy). These results are in line with previous studies that did not find a bilingual advantage in executive function, including inhibitory control (e.g., Namazi and Thordardottir, 2010; Gathercole et al., 2014; Arizmendi et al., 2018; Desjardins and Fernandez, 2018), but in contrast to many others that did find a bilingual advantage (e.g., Carlson and Meltzoff, 2008; Martin-Rhee and Bialystok, 2008; Adesope et al., 2010; Foy and Mann, 2014). At least two reasons could explain why we did not find differences between monolingual and bilingual children’s inhibitory control in the present study.

First, it is possible that the way in which bilingual children in this study used English and Spanish does not provide them with enhanced inhibitory control. Bilingual children were sequential bilinguals who learned Spanish at home and English at school (the majority language). They showed a clear dominance of English over Spanish when looking at language preference, self-proficiency ratings, and vocabulary outcomes (despite overall weekly exposure being similar). In addition, close to half of the children used English-only at home with their siblings. Therefore, bilingual children in the present study were not balanced bilinguals. Some, but not all, of the previous studies that found a bilingual advantage in inhibitory control agree that children were balanced bilinguals (e.g., Bialystok and Majumder, 1998; Yow and Li, 2015). It is possible that a balanced use of languages across contexts may provide children with the switching practice necessary for an inhibitory control advantage to emerge (Tao et al., 2011; Arizmendi et al., 2018; Nicoladis et al., 2018).

Second, although we controlled for maternal education (proxy for socio-economic status; SES) in our analyses, the fact that the majority of the bilingual children had mothers with low levels of education (high school or less) needs to be addressed more closely. Previous research has shown that SES may affect executive function (see Lawson et al., 2018 for a meta-analysis), in which higher SES is related to better executive function and that low SES can negatively affect school readiness (e.g., Micalizzi et al., 2019) and language outcomes (e.g., Hart and Risley, 1992; Noble et al., 2005; Maguire et al., 2018). When bilingual children are matched by SES with monolingual children, studies on executive function have shown conflicting results varying from a bilingual advantage (e.g., Calvo and Bialystok, 2014; Krizman et al., 2016), a bilingual advantage only for children from low SES backgrounds (e.g., Naeem et al., 2018), or no bilingual advantage (e.g., Morton and Harper, 2007). Future studies should clarify under what specific circumstances (ages, SES, tasks, use of languages, etc.), we are able to observe a bilingual advantage on executive function.

### Inhibitory Control and Vocabulary Size in Bilingual Children

According to our hypothesis, we found that English expressive vocabulary (dominant language) was related to inhibitory control in bilingual children. Spanish vocabulary (non-dominant language) was not correlated with inhibitory control. This is in line with previous studies (e.g., Bohlmann et al., 2015; Gangopadhyay et al., 2019; Kalia et al., 2019) and in contrast to Nicoladis et al. (2018). Nicoladis et al. investigated the correlations between language dominance (measured by parental report, proficiency in vocabulary tests, and knowledge of translation equivalents) and cognitive flexibility (a card sorting task) in 62 French-English bilingual children (3–7 years old) in Canada. They found that none of the measures of language dominance was correlated with cognitive flexibility. Although the authors classified children into three groups according to parental reports of language dominance (balanced, slightly dominant, and very dominant), the vocabulary scores of the children in English and French within each dominance group were very similar, and specific correlations between each vocabulary measure and cognitive flexibility were not reported in this study. It is possible that the dominant-language effect on inhibitory control found in this study is only observable in unbalanced bilinguals.

The correlations between English expressive vocabulary and inhibitory control measures were interpreted as bilingual children using their dominant language (English in this case) to self-regulate when completing the inhibition task. However, we did not ask children about their private speech or in what language they were thinking while completing the task. Our interpretation is in line with studies that found bilinguals to encode and recall information better when it is provided in their dominant language (e.g., Marian and Fausey, 2006; Francis and Baca, 2014; Bogulski et al., 2019), suggesting that there are more cognitive resources associated to the dominant language vs. the non-dominant language. The opposite direction, however, is still possible (inhibitory control predicting vocabulary). Some studies have shown that inhibitory control predicts vocabulary in the dominant language (e.g., Blom, 2019) and bidirectional relations between English expressive vocabulary and inhibitory control (e.g., Bohlmann et al., 2015). Regardless of directionality, this study showed that vocabulary in the dominant language and inhibitory control are correlated. Future studies could include bilingual children with different patterns of language dominance and examine their effects on executive function.

### Limitations

The present study only utilized one measure of executive function, the flanker task, limiting the spectrum of executive functions to a single inhibition task. Future studies should consider utilizing more than one measure of inhibitory control and other executive function measures to fully assess executive function in monolingual and bilingual children. Similarly, because language dominance may vary by language domain, future studies could include more language measures along with language exposure and use to better define language dominance.

Due to the small sample size of bilingual children included in the study, we were unable to assess what vocabulary measure was a better predictor of inhibitory control and the directionality between vocabulary and inhibitory control. We interpreted the relation between English expressive vocabulary and inhibitory control as bilingual children using their dominant language to self-regulate over their non-dominant language, increasing the inhibitory control associated to the dominant language. Our reasoning was that if inhibitory control was to be crucial for vocabulary acquisition (i.e., enhanced inhibitory control helps children learn new words and thus have larger vocabularies); this relation should be present also with vocabulary in the non-dominant language, which was not the case in this study. Yet, we were unable to test directionality in our analyses. Future studies may consider studying the directionality between inhibitory control and the dominant language in bilinguals by collecting longitudinal data.

## Conclusion

Monolingual and bilingual children did not differ in a task of inhibitory control (flanker task). In addition, vocabulary in the dominant language (English), but not in the non-dominant language (Spanish), was a significantly correlated with inhibitory control in bilingual children. This result was interpreted as bilingual children using their dominant language more frequently than their non-dominant language to process information and self-regulate, in turn increasing the inhibitory control associated with the dominant language. Future studies may consider addressing directionality to better understand the relation between inhibitory control and dominant language in bilingual children.

## Data Availability Statement

The raw data supporting the conclusions of this article will be made available by the authors, without undue reservation.

## Ethics Statement

The studies involving human participants were reviewed and approved by Arizona State University. Written informed consent to participate in this study was provided by the participants’ legal guardian/next of kin.

## Author Contributions

AS wrote the introduction, results, and discussion. BD-L collected the data, wrote the method, and contributed to the conceptualization of the manuscript. All authors contributed to the article and approved the submitted version.

## Conflict of Interest

The authors declare that the research was conducted in the absence of any commercial or financial relationships that could be construed as a potential conflict of interest.

## Publisher’s Note

All claims expressed in this article are solely those of the authors and do not necessarily represent those of their affiliated organizations, or those of the publisher, the editors and the reviewers. Any product that may be evaluated in this article, or claim that may be made by its manufacturer, is not guaranteed or endorsed by the publisher.
